# Prevalence and associated factors of infection in children with nephrotic syndrome aged 2-18 years in the northwest and east Amhara region, Ethiopia: a multi-center cross-sectional retrospective study

**DOI:** 10.1186/s12889-024-19408-7

**Published:** 2024-07-10

**Authors:** Birhanu Abie Mekonnen, Tilahun Dessie Alene, Yalemwork Anteneh Yimer, Ayenew Molla Lakew, Geta Bayu Genet

**Affiliations:** 1 Department of Pediatrics and Child Health, School of Medicine, University of Gondar, PO.Box:196, Gondar, Ethiopia; 2 Department of Pediatrics and Child Health, School of Medicine, Wollo University, Dessie, Ethiopia; 3 Department of Pediatrics and child health, School of Medicine, Bahir Dar University, Bahir Dar, Ethiopia; 4 Department of Epidemiology and Bio-statistics, Institute of Public Health, University of Gondar, Gondar, Ethiopia

**Keywords:** Serum albumin, Infection, Associated factors, Nephrotic syndrome, Children

## Abstract

**Background:**

Infection is the most common complication of pediatric patients with nephrotic syndrome. The factors associated with infection in nephrotic syndrome are lacking. The objective of the study was to identify the prevalence and associated factors among children with nephrotic syndrome aged 2 to 18 years.

**Methods:**

We conducted a hospital-based retrospective cross-sectional study. The data collector installed an Epi5 collector electronic data-collecting tool from Google Play. Then, we exported the data to Stata version 15.1 for analysis. The mean, standard deviation, frequency, and percentage were used for descriptive statistics. The logistic regression model was used to identify the factors associated with infection.

**Results:**

In this study, the prevalence of infection among nephrotic syndrome children is 39.8% (95%CI: 30.7, 49.7). The types of infection identified were pneumonia, urinary tract infection, diarrheal disease, cutaneous fungal infection, intestinal parasitic infection, and sepsis. The presence of hematuria increased the odds of infection by 5-times. On the other hand, low level of serum albumin increased the odds of infection by 7%. Being a rural resident increased the odds of infection by 3.3-times as compared to urban.

**Conclusions:**

Serum albumin level, presence of hematuria, and rural residence were significantly associated with infection. We recommended a longitudinal incidence study on large sample size at multicenter to strengthen this finding.

## Background

Nephrotic syndrome is a chronic renal disease diagnosed by the presence of nephrotic range proteinuria (first-morning proteinuria ≥ 3 + on dipstick) and either hypoalbuminemia ≤ 3 g/dl or edema when serum albumin is not available [1]. The estimated worldwide incidence of nephrotic syndrome is 4.7 per 100,000 children (range 1.15–16.9) [2, 3].

Infection is one of the complications of children with nephrotic syndrome. Other complications related to nephrotic syndrome are thromboembolism, hypovolemia, cardiovascular problems, acute renal failure, anemia, hypothyroidism, hypocalcemia, rickets/osteopenia, and intussusception [4]. The risk factors for infection in nephrotic syndrome are loss of immunoglobulins and alternative complement pathway factors B and I through urine. In addition, factors contributing to infection in NS are the presence of edema/ascites, prednisolone, and other cytotoxic agents used for its treatment [4, 5].

Studies identified that decreased levels of serum albumin and immunoglobulin were the independent risk factors or infection in children with nephrotic syndrome [6]. Low CD4^+^ T-cells and high cumulative doses of prednisolone, combined use of immunosuppressant, steroid resistance, gram positive infection were also other associated factors with severe bacterial infection in adults with nephrotic syndrome [7, 8].

The prevalence of infection in children with nephrotic syndrome ranges from 15 to 38%, including non-severe infections [9]. 15% of nephrotic syndrome patients developed severe bacterial infections (SBI) [3]. Among severe bacterial infections, pneumonia is the most common type of infection, followed by bacteremia/sepsis and UTI [2, 9, 10]. Other studies also showed that UTI was the most prevalent infection in children with nephrotic syndrome [9, 11].Spontaneous bacterial peritonitis, cellulitis, perinephric abscess, and pulmonary tuberculosis are also other types of infections in children with nephrotic syndrome [11]. In another study, about 19.6% of nephrotic syndrome patients developed episodes of major infection especially in relapse cases [12, 13]. This study will identify the prevalence and associated factors of infection among children with nephrotic syndrome, aged 2–18 years old.

## Methodology

### Study population

The study population was children aged from 2 to 18 years with the diagnosed nephrotic syndrome.

### Study design and setting

A hospital-based cross-sectional study was conducted in three tertiary hospitals in Ethiopia from 2021 to 2023. These hospitals are public hospitals and are expected to serve 20 million people. So, we selected these hospitals in order to get a sufficient sample size because they served substantial populations of patients. Pediatric emergency, inpatient wards, and outpatient follow-up services have been provided in all these hospitals. With a consecutive sampling method, 103 pediatric nephrotic syndrome patients were selected from the three comprehensive specialized hospitals. We allocated the sample through the proportional allocation sampling technique. The calculated sample size was 113 but ten of the patient chart were lost. Based on proportional allocation sampling, we obtained 48, 40 and 15 samples from University of Gondar, Dessie and Felege Hiwot Comprehensive specialized hospitals respectively. We collected the data from February 1, 2023 to June 30, 2023.

### Inclusion criteria

Children diagnosed with nephrotic syndrome who had follow up for at least 3 months.

### Exclusion criteria

Patients excluded from the study were incomplete chart and those who did not give consent via phone call. Patient with hepatitis B, C and Syphilis were excluded due to the absence of universal laboratory tests to all the study population.

### Operational definition

#### Nephrotic syndrome

Child with first morning proteinuria ≥ 3 + on dipstick and either hypoalbuminemia ≤ 3 g/dl and/or edema when serum albumin is not available [1].

#### Infection

Child with nephrotic syndrome and clinical evidence of infection documented by the treating physician.

#### Remission

Negative or trace urine dipstick on three or more consecutive occasion [1].

#### Relapse

Recurrence of nephrotic range proteinuria defined by ≥ 3 + by dipstick for three consecutive occasions [1].

#### Steroid dependent nephrotic syndrome

Two consecutive relapse during therapy with prednisolone at either full dose or tapering or 15 days of discontinuation [1].

#### Steroid resistant nephrotic syndrome

Lack of response with four weeks of daily prednisolone therapy or standard dose [1].

### Data collections procedure and variables

Medical records were retrieved by using a checklist from February 1, 2023, to June 30, 2023. We developed a checklist after reviewing the patients’ charts. Then, this checklist was constructed into the Epi5 collector application. Then, a pretest was done on six different sets of cases. One data collector from each site was assigned for data collection. Each data collector downloaded the application from Google Play and installed it on their smart phones. The principal investigators gave a brief orientation on the data collection processes and the Epi-5 collector application via Zoom.

### Data processing and analysis

The collected data was exported to Stata version 15.1 for analysis. The mean, median, standard deviation, interquartile range, and percentage were used for descriptive statistics. The model’s fitness was checked using the Hosmer and Lemeshow test, and its P-value was 0.48. Logistic regression was used to identify the factors associated with infection. A P-value of ≤ 0.05 and a 95% confidence interval were used to assess the statistical significance.

## Results

### Socio-demographic characteristics and clinical parameters

The study sample was 113. All children/guardian with nephrotic syndrome gave phone consent with 100% response rate. However, ten of the patient chart were lost. Therefore, only 103 of children with nephrotic syndrome were included in the study. The male-to-female ratio was 1.45 to 1. From the total study population, 75 (72.82%) lived in rural areas. The mean age of the study population was 8 (± 4.03 SD) years. The mean body mass index (BMI) was 16.25 (± 3.54 SD) kg/m2.

Fourteen (21.4%) of the study participants were steroid dependent, and 9.7% of them had steroid-resistant nephrotic syndrome. Around 36.89% of the study participants were in a remission state, and 32.03% were diagnosed with a relapse. The median systolic blood pressure of children with nephrotic syndrome was 110 mm-Hg, with an IQR of 100–121 mm-Hg. The median diastolic blood pressure was 70 mm-Hg, with an IQR of 65–85 mm-Hg.

Four children with nephrotic syndrome had a family history of renal disease. Three children with nephrotic syndrome had a history of bee stings before they developed nephrotic syndrome. Among the study participants, 3.9% had a self-history of atopy. Among the study participants, about 33.01% used anti-hypertensive medications. The most commonly prescribed anti-hypertensive drug by the treating physician was furosemide. The indications were hypertension in 15 (14.56%), respiratory distress in 12 (11.6%), and genital edema in seven (6.8%). Around 7.76% of the study participants had a history of non-steroidal anti-inflammatory drug (NSAID) intake **(**Table 1**).**

**Table 1:** Descriptive findings among children with nephrotic syndrome age 2-18 years attending pediatric follow-up clinic at selected referral hospitals in northwest and east Amhara region, 2022 (*n* = 103)

Variable		Frequency	Percentage
Age (year)	< 8 year	58	56.3
	≥ 8 year	45	43.7
Sex	Male	61	59.22
	Female	42	40.78
Residence	Rural	75	72.82
	Urban	28	27.18
Chief complaint	FP &HM	9	8.74
	GBS	94	91.26
Serum albumin (g/dl):	Mean = 1.904 (± 0.381SD)		
Cholesterol (mg/dl):	Mean = 396.65 ±(11.98SD)		
Triglyceride (mg/dl):	Mean = 330.59 ± (13.99 SD)		
Hematocrit (percent)	Mean = 35.35 ±(0.92SD)		
Platelet count (cell/µL)	Mean 357,728.9 ±(13,353.05SD)		
Serum Creatinine (mg/dl):	Mean = 0.74±(0.05SD)		
Proteinuria	< 3	15	14.56
	≥ 3+	88	85.44
Hematuria	Yes	57	55.34
	No	46	44.66
Hypertension	Yes	15	14.56
	No	88	85.44
HIV infection	Yes	5	4.85
	No	98	95.15
Prednisolone	Yes	32	31.1
	No	71	68.9

GBS-generalized body swelling, FP- facial puffiness, HM- hematuria

### Prevalence of infection among nephrotic syndrome children

In this study, the prevalence of infection among nephrotic syndrome children was 39.8% (95% CI: 30.7, 49.7). Among three children with nephrotic syndrome, one had any of the infections at any time in the course of the disease. The types of infections identified were pneumonia, urinary tract infection, spontaneous bacterial peritonitis, diarrheal disease, cutaneous fungal infection, intestinal parasitic infection, and sepsis. The most common types of infections were parasitic infections, urinary tract infections, pneumonia, and spontaneous bacterial peritonitis (Table 2). Children with infections were presented with different clinical presentations **(**Table 3**).** Among patients with relapse, 10/32 (31%) had an infection. In addition, of steroid-dependent nephrotic syndrome children, 9/22 (41%) had an infection. In patients with steroid-resistant nephrotic syndrome, 7/10 (70%) had an infection. Around 16% of nephrotic syndrome children had urinary casts. Among these, 12/16 (75%) had an infection. In children with infection and nephrotic syndrome, 11/32 (34.4%) were taking prednisolone.

**Table 2:** Types of infection identified in children with nephrotic syndrome attending pediatric follow-up clinic at selected referral hospitals in northwest and east Amhara region, 2022 (*n* = 103)

Types of infection	frequency	Percentage
Parasitic infection	11	10.67
Urinary tract infection (UTI)	9	8.73
Spontaneous bacterial peritonitis (SBP)	8	7.76
Pneumonia	8	7.76
Cutaneous fungal infection	6	5.83
Acute gastroenteritis	4	3.88
Sepsis	2	1.94
Others	3	2.91

**Table 3:** Attending pediatric follow-up clinic at selected referral hospitals in northwest and east Amhara region, 2022 (*n* = 103)

Clinical presentations	Frequency	Percentage
Respiratory distress	6	10.2
Cough	8	13.6
Fever	4	6.8
Decreased urine out amount	5	8.5
Skin lesion	6	10.2
Abdominal pain	8	13.6
Vomiting	4	6.8
Diarrhea	4	6.8
UTI symptoms	11	18.6
Hematuria	3	5.1

### Factors associated with infection

We used binary logistic regression to identify potential variables associated with infection in nephrotic syndrome. The variables with a p-value of < 0.2 by binary logistic regression analysis were sex, residence, serum albumin, hematuria, anti-hypertensive drug use, steroid dependence, urinary cast, total serum protein, platelet count and hematocrit. The forward conditional regression analysis was used to identify factors associated with infection. Therefore, serum albumin level, residence, and hematuria were the factors significantly associated with infection. The odds of infection were reduced by 7% with each g/dl of serum albumin increment (AOR = 0.93, 95% CI: 0.87, 0.988). Children with nephrotic syndrome from rural residences increased the likelihood of infection by 3.31 compared to urban residences (AOR = 3.31, 95% CI: 1.09, 10.1). The odds of infection were more than five times higher among patients with hematuria (AOR = 5.26, 95% CI: 2.03, 13.64) (Table 4).

**Table 4:** Factors associated with infection among children with nephrotic syndrome age 2–18 years attending pediatric clinic at referral hospital in northwest and east Amhara region, 2022

Variable		Infection		COR (95% CI)	AOR (95% CI)	*p*-value
		Yes	No			
Residence	Urban	6	22	1	1	
	Rural	35	40	3.21(1.17,8.81)	3.31(1.09,10.1)	0.035*
Serum albumin				0.92(0.87–0.978)	0.93(0.87,0.988)	0.020^*^
Hematuria	Yes	32	25	5.26(2.15–12.90)	5.26(2.03,13.64)	0.001^**^
	No	9	37	1	1	

Level of significance- * 95% CI, ** 99% CI

## Discussion

In this study of 103 nephrotic syndrome children, 41 (39.8%) had infection. Serum albumin level, hematuria and residence were factors associated with infection in nephrotic syndrome. The top four common infections were intestinal parasitic infection, urinary tract infection, spontaneous bacterial peritonitis, and pneumonia. According to research done in Delhi, India, among hospitalized nephrotic syndrome children aged 1–12 years, the incidence of major infection was 36%, and peritonitis (24%), pneumonia (18%), and UTI (15%) were the top three types of infection [14]. The other research done in India in hospitalized children aged 1–18 years indicated that the incidence of infection was 29.7%, and UTI and spontaneous bacterial peritonitis were the two common infection types(9) [15]. In addition, prospective observational research in the same country showed a 35.9% incidence of infection in children with nephrotic syndrome, and UTI (24.3%), pneumonia (21.6%), and acute diarrhea (16.2%) were the top infection types [12]. The other research done in Bangladesh, Dhaka, showed that the prevalence of infection was 38%; Urinary tract infection (46%), peritonitis (26%), septicemia (11%), and pneumonia (9%) were the four most common types of infections [11]. Both the Indian and Bangladesh studies were in line with this study’s result, in which the prevalence ranged from 31 to 50%.

In research done in Kerala, India, in children aged 1–12 with nephrotic syndrome, 19.6% of children developed infection. Of which, pneumonia (41.7%), UTI (25%), and septicemia (16.7%) were the leading types of infection [13]. The prevalence was lower in this research as compared to our study. The difference was due to the large sample size, and the study included major infections, which include only the deep organ or tissue infections that needed hospitalization. The other research done in tertiary hospitals in Israel showed that the prevalence of infection was 14.6% [10]. The prevalence in this study was lower than in our study. It was due to the exclusion of a non-severe infection in this study.

Research done in Egypt on children admitted to a nephrology unit with relapse showed that the rate of infection was 66.6%. The common types of infection identified were acute gastroenteritis (25%), UTI (21.7%), and pneumonia (15%) [16]. The prevalence of infection in this study was higher than in our study, because the study was done in children with relapse nephrotic syndrome. Relapse was important associated factor of infection in children with nephrotic syndrome. However, our study included non-relapse cases as well.

In this study, serum albumin level was associated factor of infection in children with nephrotic syndrome. It was supported by research done in China, which showed decreased serum albumin was an independent risk factor for infection [17]. It was also supported by Indian research, which indicated that a serum albumin level < 1.5 g/dl was an independent risk factor for infection in children with nephrotic syndrome [14]. The proposed mechanism of infection in children with nephrotic syndrome-related hypoalbuminemia was that hypoalbuminemia was an indicator of urinary loss of immunoglobulins and complement factors required for opsonization, phagocytosis, and host defense [14]. A study in India showed that UTI was associated with low serum albumin and high serum cholesterol level [15].

The presence of hematuria as an independent factor for children with nephrotic syndrome. Hematuria (blood in the urine) was an important clinical manifestation of a urinary tract infection. In our study, UTI was the second-leading infection type, accounting for 8.73%. In addition, among children who had UTI, 78% of them had hematuria. Research done in India showed that significant microscopic hematuria was found in 20.7% [18].

The other associated factor for infection in children with nephrotic syndrome was residence. Children in rural areas were 3.3 times more likely to have infection than children lived in urban areas. Intestinal parasitic infection accounted for 27% of the infections among children with nephrotic syndrome, and it was the leading type of infection in our study. Residence was identified as one important factor associated with intestinal parasitic infection among school-age children in Ethiopia, and living in rural areas increased the intestinal parasitic infection by fivefold [19]. In our study, children with nephrotic syndrome who developed UTI and spontaneous peritonitis were purely from rural areas. In addition, 91% of children with intestinal parasitic infections and 88% with pneumonia lived in rural areas.

The major limitation of this study was the small sample of the study population and the lack of a complete blood count of patients with infection. Although we tried to alleviate the fear of having a small sample size by including three study sites, it was not sufficient to have an adequate sample size. In addition, the data was collected from patients who had follow-ups for the last three years only. So, for other researchers who plan to research this area, it is good to incorporate more study sites and extending of study period appropriately. We recommended incorporating these limitations (complete blood count and large sample size) to have more plausible and generalizable research outcomes for future studies.

## Conclusion

In this study, we identified serum albumin level, hematuria, and residence as factors associated with infection in children with nephrotic syndrome. Infection occurred in 39.8% of children with nephrotic syndrome. The most common infections identified were intestinal parasitic infections, urinary tract infections, pneumonia, and spontaneous bacterial peritonitis. The authors recommended evaluating children with nephrotic syndrome for the presence of infection when they had low serum levels of albumin and hematuria and were from rural areas.

## Data Availability

All data generated or analyzed during this study are included in this published article.

## Abbreviations

AOR: Adjusted odds ratio
BMI: Body mass index
CI: Confidence interval
FP: Facial puffiness
GBS: Generalized body swelling
HM: Hematuria
IQR: Inter-quartile range
MCD: Minimal change disease
Mm-Hg: Millimeter of mercury
NS: Nephrotic syndrome
NSAID: Non-steroidal anti-inflammatory drugs
SBI: Severe bacterial infection
SBP: Spontaneous bacterial peritonitis
SD: Standard deviation
UTI: Urinary tract infection
